# Draft Genome Sequence of *Enterococcus faecium* Strain CRL 1879, Isolated from a Northwestern Argentinian Artisanal Cheese

**DOI:** 10.1128/genomeA.00514-13

**Published:** 2013-07-18

**Authors:** Nadia E. Suárez, Lucila Saavedra, Ivana Složilová, Julieta Bonacina, Kateřina Demnerová, Fernando Sesma

**Affiliations:** Laboratorio de Genética y Biología Molecular, Centro de Referencia para Lactobacilos (CERELA-CONICET), San Miguel de Tucumán, Tucumán, Argentinaa; Department of Dairy, Fats and Cosmetics, Faculty of Food and Biochemical Technology, Institute of Chemical Technology, Prague, Czech Republicb; Department of Biochemistry and Microbiology, Faculty of Food and Biochemical Technology, Institute of Chemical Technology, Prague, Czech Republicc

## Abstract

We report the draft genome sequence of the bacteriocin producer *Enterococcus faecium* strain CRL 1879, isolated from a northwestern Argentinian artisanal cheese. The draft genome sequence is composed of 73 contigs for 2,886,747 bp, with 3,140 protein-coding genes. Six biosynthetic clusters for bacteriocin class II production were found. Typical virulence determinants, which have relevance in food safety, were not present.

## GENOME ANNOUNCEMENT

Enterococci live as commensals of the gastrointestinal tract of warm-blooded animals, being the most abundant Gram-positive cocci in humans (1). Enterococci can also be found in foods of animal origin, vegetables, and plant materials because of their ability to survive diverse treatment and adverse environmental conditions (2). These organisms are common constituents of dairy products such as cheese, with *Enterococcus faecium* and *Enterococcus faecalis* being the most prevalent ones. Importantly, enterococci have the ability to produce enterocins, small peptides with antimicrobial properties, with potential applications as natural preservatives in the food industry (3).

Nowadays, various types of artisanal cheeses are still being produced by traditional techniques on the farms in the highlands of the province of Tucumán, Argentina. This ecological niche represents a natural source for the isolation and characterization of promising strains with biotechnological properties.

We report the draft genome sequence of *E. faecium* strain CRL 1879, a multiple-antilisterial-bacteriocin producer, isolated from an artisanal cheese (N. Suárez, J. Bonacina, I. Složilová, S. Horáčková, K. Demnerová, F. Sesma, and L. Saavedra, unpublished data).

The genomic DNA was extracted from the cultured bacterium according to the method of Pospiech and Neumann (4). The genome sequence was obtained using a whole-genome shotgun (WGS) strategy (1,240,380 total sequences; 40-fold coverage of the genome) with an Ion Torrent personal genome machine based upon libraries created using NEBNext DNA library kits (MR DNA, Shallowater, TX). Quality filtered reads were *in silico* assembled using the DNASTAR Ngen assembler, giving 73 large contigs. Genome annotation was done using the standard operating procedures (SOPs) for prokaryotic annotation from Integrative Services for Genomics Analysis (ISGA) (5) and from the Rapid Annotations using Subsystems Technology (RAST) server (6).

The draft genome sequence consists of 2,886,747 bases with a mean GC content of 37%. A total of 3,140 coding sequences (CDS) and 69 structural RNAs (61 tRNAs) were predicted. Additionally, there are 317 RAST subsystems represented in the chromosome, which represent only 45% of the sequences assigned.

Interestingly, several CDS for the production of class II bacteriocins, namely, enterocin A, enterocin B, enterocin P, enterocin SE-K4, enterocin X, and a novel two-component bacteriocin not yet described, were found.

In addition, given the importance of the genus as a potential nosocomial pathogen, a search for virulence factors associated with invasiveness and disease severity was performed (7). No *gelE* (gelatinase), *esp* (enterococcal surface protein), *ace* (enterococcal surface adhesion), *agg* (aggregation substance), or *efaA* (*Enterococcus faecalis* antigen A) genes were found, nor was *fsrB* for the *fsr* quorum-sensing system or the cytolysin operon (*cylA*, -*B*, -*M*, or *L*) (8). The genomic analysis also showed the absence of genes related to vancomycin resistance: *vanA*, *vanB*, *vanC*2, and *vanD*.

### Nucleotide sequence accession numbers.

This whole-genome shotgun project has been deposited at DDBJ/EMBL/GenBank under the accession number AOUK00000000. The version described in this paper is version AOUK00000000.1.
